# Sensors Cybersecurity

**DOI:** 10.3390/s21051762

**Published:** 2021-03-04

**Authors:** Dimitris A. Gritzalis, Grammati Pantziou, Rodrigo Román-Castro

**Affiliations:** 1 Department of Informatics Athens University of Economics & Business, GR-10434 Athens, Greece; 2 Department of Informatics & Computer Engineering, University of West Attica, 12241 Athens, Greece; pantziou@uniwa.gr; 3 Department of Computer Science, University of Malaga, 29071 Malaga, Spain; roman@lcc.uma.es

At present, sensors are increasingly used in all kinds of platforms, manned or unmanned, particularly in view of the emerging Internet of Things (IoT). The quality and capabilities of drones, wearables, driverless cars, sensor-aided shipping and aviation, robots, and any other sensor-based platform or application mainly depend on the sensing technology they use. No single type of sensor can provide solutions to all problems, and most of them are vulnerable to cyberattacks. For example, host-based sensors provide more extensive and accurate information, but this only applies to phenomena that they are manufactured to describe. From their side, network-based sensors provide extensive coverage, but they can be deceived by traffic engineering. Further, they cannot describe encrypted traffic and often can hardly estimate the activity of a host. In addition, because of the wireless- and resource-constrained nature of sensor networks and their possible deployment in harsh environments or unattended areas, they are susceptible to many security threats. Therefore, sensor-based platforms and applications are highly vulnerable to cyberattacks, and many valuable assets are exposed and vulnerable to sensor and sensor network-based cyber threats. As a result, the need for adequate sensor and sensor network infrastructure protection and the development of secure information monitoring systems is increasing. However, due to the communication, computation, and delay constraints of sensor networks and applications, achieving an acceptable level of security has been a difficult issue to solve. Therefore, we need to develop new theories, technologies, and practical solutions so as to protect the network infrastructure and the sensors that are integrated in the platforms of drones, mobile phones, cars, vessels, airplanes, robots, critical infrastructures, etc. From their side, the developers of secure information monitoring systems should take into account that these systems are based on data collected from multiple and often heterogeneous sensors that generate different types of data. Developing a secure sensor system requires balancing its completeness and redundancy while ensuring adequate protection against cyberattacks. 

This editorial presents the manuscripts accepted, after a careful peer-review process, for publication in the Special Issue “Sensor Cybersecurity” of the MDPI journal *Sensors*. This Special Issue includes seven articles: five original research papers describing original ideas, results, and real-world experiences involving sensors and cybersecurity and two review papers focusing on cyber security issues in two application areas involving sensor technology: the Internet of Medical Things and the defense of critical infrastructure from drone attacks. 

The first review paper [1] focuses on the security issues in the Internet of Medical Things (IoMT) communications. The IoMT is an interconnected infrastructure of medical systems, sensors, and services that enables wireless and remote medical devices to securely communicate over the Internet, in order to allow medical data analysis and to support real-time, remote patient monitoring and treatment. Given the sensitivity of health information, the security of the collection, storage, and processing of data is the first concern in the IoMT. In addition, data integrity and the confidentiality and availability of the medical data should be ensured. The authors of [1] present a taxonomy of IoMT-specific communication protocols as well as their inherent security characteristics, weaknesses, and relevant attacks. In addition, real case attack scenarios against medical devices are discussed and, based on these use case scenarios, a suitability assessment of the aforementioned communication protocols is provided. Finally, the authors present open issues and challenges for IoMT protocol security.

Unmanned aircraft systems (UAS) are used to support search and rescue operations, monitor and assess critical infrastructure, contribute firefighting operations, etc. However, UAS can also be used by actors with specific objectives for malicious schemes, such as aerial attacks against airport facilities and other critical infrastructures. Counteracting technologies, risk management, and resilience plans are needed for protecting critical infrastructures from such attacks. In [2], a survey is presented of drone incidents threatening airport facilities and critical infrastructure, as well as a literature review of sensor technologies capable of identifying, preventing, and mitigating rogue drone activity. The authors present the benefits and limitations of available counter-drone technologies (C-UAS). In addition, three realistic scenarios of malicious drone attack are presented, and in each case an effective C-UAS protection plan is proposed. Finally, the authors highlight the restrictions on the applicability of C-UAS to the aviation context and propose a resilience action plan for airports to defend against threats from UAS misuse.

A Trusted Execution Environment (TEE) is an implementation of the TEE standard which creates an isolated secure environment functioning in parallel with the operating system. TrustZone-based TEEs utilize both hardware and software to protect data. They have been utilized for the implementation of security-oriented solutions for smart connected devices. Although TEEs were proposed as a reliable security approach, existing published attacks against widely used TEE implementations imply that a review of their security is required. The aim of paper [3] is to provide a detailed exploration of TrustZone-based TEE vulnerabilities in order to identify design and implementation defects. To this end, the authors provide a classification of TrustZone attacks, analyze them, and make a number of critical remarks about their nature. They also provide a critical evaluation of the vulnerabilities to identify the underlying causes, which mostly include the closed implementations, the lack of security mechanisms, and the lack of tools to audit trusted applications. The authors conclude that although TrustZone technology provides all the necessary tools in order to create a secure environment for next-generation IoT networks and applications, it is still immature. They also discuss possible solutions to the identified issues that could be adopted by TEE implementers to correct and improve TrustZone’s security attitude and provide future research directions.

In paper [4], the authors study the problem of selective routing attacks in Wireless Sensor Networks. They propose an intrusion detection system (IDS) to deal with a new type of attack, where a node can be falsely accused of being malicious if its upstream node behaves maliciously. This thread is called an upstream-node effect and limits the accuracy of monitoring functions in deciding whether a node is malicious or not. The proposed intrusion detection scheme is a one-dimensional one-class classifier, called relaxed flow conservation constraint, that uses a threshold to identify normal packet loss and packet loss due to attacks. To monitor their neighbors, the nodes apply three relaxed flow conservation constraints using one-hop knowledge and one constraint using two-hop information. The two-hop information is obtained using the two-hop energy-efficient reporting scheme together with security mechanisms, such as authentication, encryption, filtering of ratings, and isolation. The authors analyze the security of the two-hop reporting scheme and its resilience against potential attacks, such as attacks against the key management system, attacks against the authentication mechanism, attacks using fabrication, and the beacon dropping attack. They also provide a theoretical analysis of the full-resilience probability of the proposed system against selective routing attacks and unfair ratings. The performance of the proposed intrusion detection system against selective routing attacks is studied under the communication complexity and message complexity metrics. The results show that the proposed classifier has a low computational cost, is appropriate for networks that are operating under quasi-stable conditions in terms of link quality, and the energy consumption is insignificant. Finally, the simulation results for the IDS performance against selective routing attacks using the GloMoSim simulator show that the proposed system achieves good results in terms of detection effectiveness.

Despite the technological developments in IoT, in particular with regard to connectivity and networking, the cyber security aspects of IoT systems have not been adequately addressed, and there is a lack of IoT testing and training facilities that focus on security. To address this shortcoming, the authors in [5] present an IoT Cyber Range (IoT-CR), which is a user-focused IoT testbed designed for IoT security training and research. IoT-CR allows users to define and work on customizable IoT networks. It supports multiple users and the simultaneous execution of multiple scalable scenarios following a modular architecture consisting of a front end and a back end that are loosely coupled via a RESTful API. The user submits for execution their scenario that includes the network topology, the configuration, and the IoT application, and the system provides them with log files detailing the emulation. The paper provides a proof-of-concept via scenarios demonstrating typical blue team/red team cyber security events and involving a variant of man-in-the-middle attack using IoT devices.

The interconnected nature of Industry 4.0, the pace of digital transformation, and the advent of cloud services mean that cyberattacks can have more far-reaching effects than ever before, and manufacturers need to be well prepared to adequately address cyber threads. Cybersecurity strategies must be secure and resilient and fully integrated into the organizational and information technology processes from the outset. To achieve cybersecurity, organizations must perform risk management, and the most fundamental component of risk management is risk assessment. Risk assessment has the purpose of identifying threats and calculating their risk levels, allowing organizations to determine or update their cyber risk strategies. Risk assessment is performed in three tiers: the organizational tier, the business process tier, and the information systems (IS) tier. In [6], a new approach is proposed for automated risk estimation in smart sensor environments at the IS tier (IS-related risk), called ARES. Organizations can use ARES to identify the assets operating under the business process together with the relevant risks. In this way, organizations can conduct a risk assessment according to their business needs and be protected against threats. In addition, a computer-assisted procedure is proposed for mapping attack patterns-to-platforms. 

Existing research work on detecting phishing attacks suggests data-driven approaches that use supervised or unsupervised machine learning under single-layered detection models. Their feature selection approach focuses on the extraction of domain characteristics that could be tampered with by adversaries. Additionally, the feature extraction strategies require large amounts of computational processing, single-layered approaches are constrained by high computational requirements and therefore cannot be effectively implemented in a real production environment. In [7], a multi-level system is proposed where feature costs can be prioritized for uncertain classifications, so that computational power is saved. In particular, the authors present a framework for detecting active fishing attacks that follows a two-layered approach to identify fishing domains based on supervised machine learning. The resource-demanding operations of the second layer can be avoided based on the prediction confidence of the first inexpensive detection layer. Thus, infrastructure resources are saved and the framework can be more effectively implemented in a production environment, while achieving a comparable accuracy with previous approaches based on single-layer supervised machine learning. The authors implemented and evaluated the performance of the framework on a dataset consisting of active phishing attacks.
